# Comparison of TEVA vs. PRAAT in the Acoustic Characterization of the Tracheoesophageal Voice in Laryngectomized Patients

**DOI:** 10.3390/jcm13133748

**Published:** 2024-06-27

**Authors:** Alejandro Klein-Rodríguez, Irma Cabo-Varela, Francisco Vázquez-de la Iglesia, Carlos M. Chiesa-Estomba, Miguel Mayo-Yáñez

**Affiliations:** 1 Otorhinolaryngology—Head and Neck Surgery Department, Complexo Hospitalario Universitario (A Coruña (CHUAC), 15006 A Coruña, Spain; alejandro.klein.rodriguez@sergas.es (A.K.-R.); irma.cabo.varela@sergas.es (I.C.-V.); francisco.vazquez.de.la.iglesia@sergas.es (F.V.-d.l.I.); 2 Otorhinolaryngology-Head and Neck Surgery Research Group, Institute of Biomedical Research of A Coruña (INIBIC), Complexo Hospitalario Universitario de A Coruña (CHUAC), Universidade da Coruña (UDC), 15006 A Coruña, Spain; 3 Health Sciences Programme, International Center for Doctorate (EIDUDC), Universidade da Coruña (UDC), 15001 A Coruña, Spain; 4 Otorhinolaryngology—Head and Neck Surgery Department, Hospital Universitario Donostia—Biodonostia Research Institute, 20014 Donostia, Spain; chiesaestomba86@gmail.com; 5 Otorhinolaryngology—Head and Neck Surgery Department, Hospital San Rafael (HSR), 15006 A Coruña, Spain

**Keywords:** larynx, tracheoesophageal puncture, speech, analysis, software, laryngectomy, rehabilitation, prosthesis

## Abstract

**Background:** Previous studies have assessed the capability of PRAAT for acoustic voice analysis in total laryngectomized (TL) patients, although this software was designed for acoustic analysis of laryngeal voice. Recently, we have witnessed the development of specialized acoustic analysis software, Tracheoesophageal Voice Analysis (TEVA). This study aims to compare the analysis with both programs in TL patients. **Methods:** Observational analytical study of 34 TL patients where a quantitative acoustic analysis was performed for stable phonation with vowels [a] and [i] as well as spectrographic characterization using the TEVA and PRAAT software. **Results:** The Voice Handicap Index (VHI-10) showed a mean score of 11.29 ± 11.16 points, categorized as a moderate handicap. TEVA analysis found lower values in the fundamental frequency vs. PRAAT (*p* < 0.05). A significant increase in shimmer values was observed with TEVA (>20%). No significant differences were found between spectrographic analysis with TEVA and PRAAT. **Conclusions:** Tracheoesophageal speech is an alaryngeal voice, characterized by a higher degree of irregularity and noise compared to laryngeal speech. Consequently, it necessitates a more tailored approach using objective assessment tools adapted to these distinct features, like TEVA, that are designed specifically for TL patients. This study provides statistical evidence supporting its reliability and suitability for the evaluation and tracking of tracheoesophageal speakers.

## 1. Introduction

The PRAAT software represents a prominent tool in contemporary objective acoustic analysis. Developed by Boersma and Weenik, this software enables the comprehensive analysis, synthesis, and manipulation of acoustic signals related to vocalization, achieved through the systematic adjustment of various parameters for the extraction of speech data and the evaluation of vocal quality [1]. PRAAT is a program designed especially for research in phonetics and to offer a tool to carry out general acoustic analysis of voice and speech as well as to use it for educational purposes.

Normally, the program is used under laryngeal speech conditions with rhythmic, periodic, and harmonic vocal fold movements.

The loss of vocal capacity in laryngectomized patients is a significant consequence of the total removal of the larynx, a critical structure for speech production. This surgical procedure compelled the laryngectomized patients to seek alternatives, such as tracheoesophageal speech (TES) or the use of voice prostheses (VP), to regain verbal communication that would lack the harmony and periodic vibration produced by the vocal folds [2]. The loss of natural voice is a complex emotional and functional transition for these patients, and vocal rehabilitation plays a crucial role in their adaptation to this new reality. The quality of voice in total laryngectomized (TL) patients relying on TES is intrinsically tied to the characteristics of the neo-glottis. Variability in the functioning of the neo-glottis and the vibration of the pharyngoesophageal tract following medical interventions, including surgery and radiation therapy (RT), results in substantial disparities in speech intelligibility and quality [3,4].

Previous studies have assessed the capability of PRAAT for acoustic voice analysis in TL patients. However, due to the subjective differences and phonatory physiology variances between patients with a larynx and those without, these findings appear to be less representative of vocal acoustics research [2,5]. For this reason, recent years have witnessed the development of specialized acoustic analysis software, such as Tracheoesophageal Voice Analysis (TEVA) [6]. This program was conceived to support the education, rehabilitation, and research endeavors of professionals working with TES and to benefit patients employing VP. The TEVA software is an integrated component within the phonetic analysis platform PRAAT, built upon the acoustic analysis framework outlined by Van As-Brooks, which categorizes voices based on phonation stability, duration, and the presence or absence of harmonics [3,7].

The difficulty of studying the speech of LT patients lies in the instability and irregularity of phonation, which is why precise and individualized calibrations and adjustments are required to obtain reliable results.

In acoustic signal typing, the voice characteristics are determined using acoustic analysis of speech. The typing is based on both visual inspection of plots of these analysis parameters and quantitative measures of a short (if possible, at least 2 s long) stretch of “stable” speech.

TEVA is a tool based on PRAAT software and designed for the specific analysis of patients with TES. Currently, there are not any studies comparing both analysis programs in laryngectomized patients. The aim of the study is to compare both acoustic analysis software in an attempt to find distinctions that will increase knowledge about transesophageal voice and thus improve education and rehabilitation for this type of patient.

## 2. Materials and Methods

### 2.1. Study Design

An observational analytical cohort study of TL patients recruited consecutively from the outpatient otorhinolaryngology clinics of a tertiary university hospital as they attended to routine follow-up from February 2019 to 2022. All patients were informed and invited to participate. The study was approved by the hospital’s ethics committee (2022/094) and informed consent was obtained in all cases.

The objective of the study was to know if the results obtained with the TEVA program compared with PRAAT were more similar to what was initially expected. We would expect to find lower fundamental frequency values, as tracheoesophageal voices apparently subjectively seem to have. Also, with a higher frequency and amplitude variation component (jitter and shimmer). Apparently, at the beginning of the process, the differences in intensity should not be very striking between both programs.

### 2.2. Inclusion and Exclusion Criteria

The study exclusively involved voluntary participants who were users of Provox Vega^®^ and met specific eligibility criteria.

The Provox Vega^®^ prosthesis is a silicone device with a double flap that is placed on the tracheoesophageal wall. This prosthesis allows the passage of air from the trachea to the esophagus in a unidirectional way, so that the air vibrating in the pharyngoesophageal tract and the neopharynx generates the voice that is subsequently modulated and articulated in the oral cavity. Additionally, the valve prevents the passage of food or liquids from the esophagus to the trachea.

These criteria included being aged 18 or older, having undergone a total laryngectomy at least 3 months prior, having completed radiotherapy or chemotherapy (if applicable) at least 3 months ago, receiving treatment with proton-pump inhibitors, and having at least 3 months of experience using the Provox Vega^®^. Individuals were excluded from participation if they had medical conditions that prevented them from using the Provox system, had recurrent or metastatic diseases, had undergone a total or partial pharyngectomy, utilized alternative phonation methods instead of a voice prosthesis, experienced functional incapacity to independently maintain the voice prosthesis, or had impaired cognitive abilities. The presence or type of cervical dissection, as well as the type of tracheoesophageal puncture (primary or secondary), did not constitute an exclusion criterion.

The patients included had received phonatory rehabilitation from speech therapy. They had a pre-surgical evaluation, and later, after the intervention, they were evaluated even before discharge, normally 10 days after surgery. After discharge, the phonatory rehabilitation work continued in the speech therapy consultation.

In total, 47 patients were invited to participate; 6 patients who met the criteria refused to participate in the study because they did not want to participate in it. The main reason for not wanting to be included was the extension of the consultation by approximately 30–45 min to carry out the speech study.

Finally, 34 patients who fulfilled the criteria were included in the study.

At the time of the assessment, seven patients did not use the Provox System, which is the reason why they were excluded from the study. In all of these patients, a tracheoesophageal puncture (1st or 2nd) was performed, but due to complications related to it (mainly wide phonatory fistula with extrusions or ingestions of the prosthesis), it was finally decided to close the tracheoesophageal fistula, so at the time of assessment in consultation, it was not possible to include them in the study since the speech with the tracheoesophageal voice could not be recorded.

### 2.3. Collected Variables

Throughout the study, every patient received an anterograde voice prosthesis (VP) insertion, and their speech was evaluated while they manually occluded the stoma using a heat and moisture exchanger device. The assessment of the patients was carried out by both an otolaryngologist and a speech therapist. The following descriptive variables were recorded: age, months since surgery, complementary treatment with RT, primary or secondary tracheoesophageal puncture (TEP), number of VP (model), number of VP replacements, presence of pulmonary pathology or concomitant swallowing problems, and pharyngeal closure technique.

A perceptual analysis with the GRBAS scale (grade, roughness, breathiness, asthenia, strain) was carried out after reading a fragment of “Platero y Yo” (J.R. Jiménez) and the validated and adapted questionnaire Voice Handicap Index 10 (VHI-10) in Spanish [8].

The GRBAS was assessed on a 4-point scale (0 = normal, 1 = slight, 2 = moderate, 3 = extreme). The numbers included in the results are the total addition of the 5 variables included (GRBAS).

The included text is a fragment of “Platero y yo”. It consists of 104 words with important phonetic richness and is widely used in subjective evaluations of the voice in the Spanish language.

The patient was recorded on 3 occasions, with the examiner selecting the best of the 3 attempts and evaluating the GRBAS scale at that time.

The assessment is carried out by two examiners independently, comparing one by one the results obtained for each item of GRBAS. If there are differences between the data recorded by each examiner, it is evaluated jointly to reach a common value according to the arguments of each one in a consensual way.

A subjective visual and acoustic adjustment was performed by viewing the spectrogram of the most stable segments of speech, lasting at least two seconds if possible. After this adjustment, the quantitative acoustic variables investigated were average sound pressure level (SPL) (dB), maximum SPL (dB), fundamental frequency (F0) Hz, jitter frequency disturbance, shimmer amplitude disturbance, and harmonic to noise ratio (HNR) for stable phonation of at least 2 s with vowels [a] and [i].

The variables were analyzed automatically with both of the software algorithms, and the numerical values were recorded for each of them.

The narrowband spectrographic characterization was developed according to spectrogram criteria [3].

The acoustic analysis was carried out using the NKI TE-Voice Analysis tool (TEVA) E5C4E4ADC5 2015-05-11T10:38:35Z software version and the PRAAT^®^ 6.1.08 software version on a Hewlett-Packard computer (Intel^®^ Core™ i5-4570SCPU 2.90Ghz) with an EliteDisplay^®^ E231 monitor and Condenser^®^ SF-666 microphone.

### 2.4. Recording Environment

All participants were recorded under the same conditions in a 4 × 3 m soundproof room with the Condenser^®^ SF-666 microphone.

The microphone was calibrated with the PRAAT program, first performing phonation as soft as possible, with a whisper, and then performing phonation with a sustained vowel at a higher pitch, checking in the PRAAT sound recorder that the sound meter was not present at too high a threshold (red/yellow color). If it is yellow or red, the microphone was moved a little further away from the mouth to avoid a high component of noise [9]. The angle between the microphone and the mouth was around 45°, and the distance between them was 5–10 cm [9].

Finally, the SPL measurement was carried out using a mobile app (Niosh Sound Level Meter App). The mobile phone with the SPL app was placed about 30 cm in front of the mouth, and it is displayed to show how many dB the device measures, comparing with the result that was marked in the PRAAT of the same person performing a phonation with the phoneme [a] in the usual tone and, if possible, for a duration of 5 s. It was recorded in PRAAT and compared with the dB of the mobile app measurement. Adjusting the difference in dB obtained between both (adding or subtracting the dB that differs between the PRAAT and the SPL meter, taking the SPL meter as a more reliable reference).

Three attempts were made to record each phoneme [a] and [i], emitting a phonation for as long as possible in the usual conversational tone. The best of the 3 recordings was selected for each phoneme based on the existence or not and stability of a pitch curve, the dispersion of the formants, the noise, and the distribution of the pulses in the spectrogram. Visualizing these characteristics of regularity in the path of the sound wave, the 2 s of the recording that showed the most stable parameters, with less noise and dispersion of the sample, were identified, which were those that were included as a study sample to carry out the instrumental study.

Signal typing was categorized based on the visual characteristics observed in the narrowband spectrogram.

For TEVA, there are three options to adjust the pitch: a low and high pitch cutoff (300 and 600 Hz). We took a high-pitched cutoff (until 600 Hz). For PRAAT, we use a manual range of 30–600 Hz.

For the selection of vocal fragments for vocal analysis, the most stable parts were chosen, >2 s, with the largest component of visible, clear harmonics.

For the spectrographic analysis for both programs, the visualization of the narrow band spectrogram was carried out based on the Yanagihara classification [10].

- Type I: The regular harmonic components are mixed with the noise component, chiefly in the formant region of the vowels.
- Type II: The noise components in the second formants predominate over the harmonic components, and slight additional noise components appear in the high-frequency region above 3000 Hz.
- Type III: The second formants are totally replaced by noise components, and the additional noise components above 3000 Hz further intensify their energy and expand their range.
- Type IV: The second formants are replaced by noise components, and even the first formants of all vowels often lose their periodic components, which are supplemented by noise components. In addition, more intensified, high-frequency additional noise components are seen.

The detection of the formants was carried out one by one, and a manual adjustment of the phonatory intervals was made with greater stability and with more horizontal tracings in the spectrogram.

Above all, the difficulty was in cases in which the first formant was close to F0.

In TEVA, for example, a stable [a] sound will show a smooth spectrogram with many harmonics as horizontal lines. The more harmonics are clearly visible, the better the voice is.

### 2.5. Statistical Analysis

Statistical analysis was conducted using Stata 14.2 for Windows (StataCorp, LLC., College Station, TX, USA). Two-tailed statistical tests were employed, and a 95% confidence interval was utilized. Normality was assessed through the Kolmogorov–Smirnov test, while variances were examined using the Levene test. Quantitative variables were presented as mean ± standard deviation (SD) and median when applicable. Group mean comparisons were carried out using the Student’s *t*-test, Mann–Whitney test, ANOVA, or Kruskal–Wallis test as appropriate. Qualitative variables were represented as frequency and percentage. Group differences were assessed through the chi-square test, Fisher’s exact test, or their respective variants when suitable.

## 3. Results

### 3.1. Descriptive Analysis

A total of 34 patients were included (Table 1). All were men with a mean age of 63.41 ± 9.55 years (Table 2). With regard to the type of surgery performed, the most frequent intervention was TL with bilateral neck dissection in 21 patients (63.6%). The most frequent tumor locations were the glottis and supraglottic areas, with 20 patients affected (10 in each location, 30.3%, respectively), followed by the transglottic in 8 patients (24.2%). The pathological TNM in most cases was advanced stages T3–T4, with 30 patients (85.7%). The most commonly performed pharyngeal suture technique was a T-closure, to which a Tapia corset was added in 12 patients (35.30%), followed by a Hormaeche closure in 10 patients (29.4%). The remaining 12 patients (35.30%) were classified as others (including the association of the T-closure with other techniques, such as discontinuous closure, closure over a salivary tube, or a microvascular flap). More than half of the patients (n = 20, 58.8%) received adjunctive treatment with RT, with a mean of 54.62 Gy ± 4.43.

A primary TEP was performed in 20 patients (58.8%) and a secondary TEP in 14 patients (41.2%). In most cases of secondary TEP, a previous primary TEP was performed (71.42%). The second intervention was due to local complications of the TEP or its closure. The number of the VP placed at the moment of study in most cases was Provox No. 8 (50%). The second most frequent was Provox No. 6 (32.35%).

### 3.2. Vocal Analysis

#### 3.2.1. Subjective Analysis

The mean score of the GRBAS scale was 7.35 ± 3.35. Self-perception by the patients, evaluated with the Voice Handicap Index (VHI-10) test, showed a mean score of 11.29 ± 11.16 points, categorized as moderate handicap (Table 2).

#### 3.2.2. Acoustic Analysis

The acoustic analysis comparing the TEVA and PRAAT programs for the phonemes [a] and [i] is summarized in Table 3. In all cases, differences were found in the acoustic analysis results between TEVA and PRAAT, except for Shimmer [a].

In the acoustic analysis of the [a] phoneme, the jitter analysis obtained an average of 2.09% with TEVA and 2.86% with PRAAT.

In the variation component of the amplitude studied with the shimmer of the [a] phoneme, the value is larger with the TEVA (mean 25.6%) compared to the PRAAT, which obtained a mean value of 15%.

The mean and maximum [a] frequencies have similar values, especially in the results of average intensity (TEVA 64.1 dB vs. PRAAT 63.9 dB). In the case of the maximum intensity, there was a greater difference (TEVA 74.8 dB vs. PRAAT 66.9 dB).

The mean fundamental frequency differs between both softwares, with the average in the analysis with TEVA being 105 Hz and with PRAAT being 275 Hz.

Finally, regarding [a] HNR, results with similar figures were obtained with both softwares (TEVA 3.36 dB vs. PRAAT 3.49 dB).

Regarding the acoustic analysis of the [i] phoneme, in this case, in the jitter value, there is a greater difference in the obtained result (TEVA 1.57% vs. PRAAT 2.8%).

The result of shimmer in the [i] phoneme, as occurred in the [a] analysis, is higher with the TEVA analysis, 22.4% vs. PRAAT 14.9%.

In the case of [i], the average and maximum intensities are much greater, even TEVA at 63.3 dB and 66.9 dB (mean and maximum, respectively) vs. PRAAT at 62 dB and 64.8 dB.

In relation to the fundamental frequency, statistically significant differences were also found (TEVA 109 Hz vs. PRAAT 142 Hz).

Finally, the [i] HNR was higher with TEVA at 5.46 dB vs. PRAAT at 4.56 dB.

**Figure 1:**
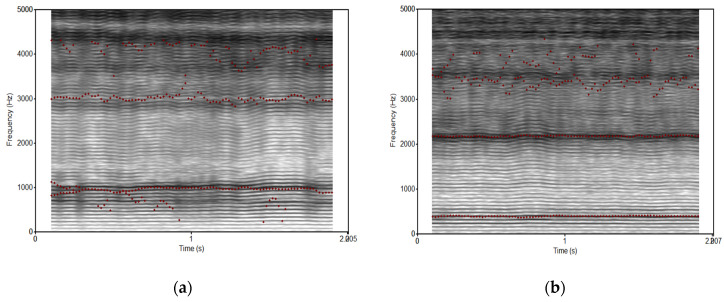
TEVA spectrographic analysis of [a] and [i]. Manually adjusted and selected sample in a sustained vowel of two-second duration. The stability and sharpness of the harmonics and formants are appreciated. (**a**) [a] Phoneme; (**b**) [i] phoneme.

Regarding the spectrographic analysis, no significant differences were found between both softwares, TEVA vs. PRAAT (Figure 1 and Table 4).

## 4. Discussion

Acoustic signal typing and analysis is used in laryngeal voice and is often recorded with PRAAT software [11,12]. However, standard acoustic voice analyses are not always suitable to measure substitute voices because speech originating in the vibrating pharyngo-esophageal segment, as TES, is known to contain more noise components and less regularity than laryngeal voice [2,13]. Therefore, the acoustic analysis of the tracheoesophageal voice continues to be a challenge today. Specific programs have been developed for TES [3,4]. This involved categorizing tracheoesophageal voices into four subtypes based on visual assessment of the acoustic content of narrow-band spectrograms supported by written guidelines [3,14,15].

Most of the recent studies demonstrate the superiority of the results obtained from instrumental and subjective acoustic analysis in patients using VP compared to other types of phonatory rehabilitation [16]. Recent reviews also demonstrated the best results in subjective questionnaires analyzing vocal quality, intelligibility, and quality of life [17].

Despite TEVA being a tool based on PRAAT, there are currently no studies comparing both analysis methods in laryngectomized patients. This study aims to conduct a comparative analysis of both objective acoustic analysis programs, with the objective of discerning any distinctions that may determine the suitability of one program over the other for the investigation of phonatory quality in TL patients.

In relation to the results obtained in our study in the VHI survey, an average of 11.29 was observed, classified as moderate handicap, but very close to a mild handicap value (less than 10 points) [18]. These results demonstrate the great satisfaction of patients with their speech rehabilitation method. The lower values in the fundamental frequency with the TEVA analysis are notable, considering that they are subjectively perceived as deep voices; therefore, these results are more in line with reality.

Regarding the speech stability values, the jitter evaluates the variation of the F0 between one cycle and the next [8,18,19]. The adaptation of the acoustic study to TES patients with TEVA demonstrates values closer to normalcy (<1%) for both phonemes [a] and [i] (2.09% and 1.57%, respectively) compared with the study with PRAAT (2.86% and 2.8%). Another equilibrium value is the shimmer parameter, which evaluates the variability of the amplitude from cycle to cycle and is inversely related to vocal intensity. For laryngeal voice (normal shimmer value <7%), the speech intensity during conversation is between 75 and 80 dB and depends on variables such as subglottal pressure, glottal closure, and respiratory capacity [18]. TL patients lose their laryngeal functions, and the airflow regulation needed for speech emission is worse. Furthermore, these patients, with a history of a smoking habit in most cases, usually have smaller lung capacities because of their respiratory pathology. For this reason, TL patients have intensity numbers lower than normal, considerably increasing shimmer values.

Despite the differences observed in our study in the formant analysis carried out with TEVA and PRAAT, no statistically significant differences were found, as were expected. It is worth noting the higher number of patients in III and IV grades for the [a] phoneme with TEVA (29.4%) than with PRAAT (11.7%). The same occurs for the phoneme [i] (TEVA n = 11; 32.3% vs. PRAAT n = 6; 17.6%). This fact can be explained because with TEVA, a more specific TES analysis is carried out, in which a less stable voice is identified and has fewer harmonic components than in the spectrographic analysis with PRAAT [3].

Tracheoesophageal speech (TES) seems to be more comparable to healthy speech, but it is necessary to go one step further to more precisely categorize this type of voice [2,7,11,12,13,14]. Therefore, analysis with a specific program for TES, such as TEVA, is more suitable for voices with VP since the adjustment of parameters for alaryngeal voices with a greater noise component is more accurate.

In the statistical analysis, significant results were observed in most of the variables. This means that the differences are not simply due to chance, and there are changes or differences between the two.

The difference between both programs that makes us think that TEVA is more specific and better adjusted to the characteristics of TES patients is that it categorizes the TES voices based on Van As-Brooks classification, keeping in mind the specific phonation stability, duration, and the presence or absence of harmonics in these kinds of voices.

On the other hand, PRAAT is a program designed for the analysis of laryngeal voices, with many of its algorithms focused on the fundamental frequency, which is much more irregular in patients with TES, so the analysis of these voices may be less adjusted to reality.

That is why, with exactly the same samples being studied, the TEVA software seems more adapted to the characteristics of laryngectomized patients who use TES. Furthermore, the results obtained with the TEVA are more similar to those expected initially, as we have commented before, especially in terms of fundamental frequency and amplitude variation.

The acoustic analysis was performed by individualizing each patient and adjusting the study to obtain the different variables in the most reliable way possible. For this reason, we believe that the values are as correct as possible.

## 5. Conclusions

In our study, the instrumental acoustic variables comparing the PRAAT and TEVA programs demonstrate with statistically significant evidence that the TEVA program could adjust more precisely and reliably to patients with alaryngeal voices who use TES. The differences observed between both types of software may be due to a better adjustment of the automatic parameters in the TEVA, and both may be complementary to categorize these types of voices in a more complete way. Furthermore, it would be interesting to expand the study sample, including patients of both genders, and try to improve the acoustic signal typing and objective vocal analysis of this type of patient.

## Figures and Tables

**Table 1:** Descriptive analysis.

Variables	Subgroups	N (%)
**Surgery Type**	TL ^1^ + BFND ^2^	21 (63.6)
	TL + FLND ^3^ + RRND ^4^	1 (3)
	TL + BFND + RFFF ^5^	2 (6.1)
	TL + BFND	3 (9.1)
	TL + RLND ^6^ + FRND ^7^	2 (6.1)
	TL + FLND	3 (9.1)
	TL + RRND	1 (3)
**Tumor location**	Transglottic	8 (24.2)
	Supraglottic	10 (30.3)
	Glottic	10 (30.3)
	Hypopharynx	5 (15.2)
**pTNM**	T4N1	2 (7.1)
	T3N1	2 (7.1)
	T4N0	3 (10.7)
	T2N0	4 (14.3)
	T3N2	10 (35.7)
	T3N0	6 (21.4)
	T4N2	1 (3.6)
**TEP ^8^**	Primary	20 (58.8)
	Secondary	14 (41.2)
**N. of voice prosthesis**	8	17 (50)
	6	11 (32.4)
	4	5 (14.7)
	10	1 (2.9)
**Radiotherapy treatment**	Yes	20 (58.8)
	No	14 (41.2)
**Pulmonary pathology**	Yes	11 (32.4)
	No	23 (67.6)
**Dysphagia**	No	34 (100)
**Pharyngeal closure**	T + Tapia corset	12 (35.3)
	Hormaeche	12 (35.3)
	Others	10 (29.4)

^1^ TL, total laryngectomy; ^2^ BFND, bilateral functional neck dissection; ^3^ FLND, functional left neck dissection; ^4^ RRND, radical right neck dissection; ^5^ RFFF, radial forearm free flap; ^6^ RLND, radical left neck dissection; ^7^ FRND, functional right neck dissection; ^8^ TEP, tracheoesophageal puncture.

**Table 2:** Descriptive analysis of continuous variables, subjective characterization of TES, and formants of [a] and [i] with PRAAT.

	Mean	Median	Standard Deviation	Min	Max
**Age (years)**	63.41	62.50	9.55	48.00	89.0
**Number of VP ^1^ replacements**	4.12	3.00	3.68	1.00	17.0
**Gy ^2^ of RT ^3^ received**	54.62	54.00	4.43	46.00	64.0
**GRBAS ^4^**	7.35	7.50	3.36	1.00	14.0
**VHI ^5^**	11.29	6.50	11.16	0.00	34.0
**[a] Phonation time (s)**	13.16	13.13	5.89	3.20	24.3
**[a] 1° formant Frequency (F1)(Hz)**	835.42	826.84	118.08	627.49	1059.1
**[a] 2° formant Frequency (F2)(Hz)**	1560.44	1553.92	209.94	1205.41	2272.2
**[a] 3° formant Frequency (F3)(Hz)**	2955.33	2948.03	242.54	2429.03	3477.7
**[i] Phonation time (s)**	12.11	11.15	5.84	2.89	25.6
**[i] 1° formant Frequency (F1)(Hz)**	472.86	388.39	210.93	283.26	1074.5
**[i] 2° formant Frequency (F2)(Hz)**	2450.03	2454.26	211.79	1838.55	2826.2
**[i] 3° formant Frequency (F3)(Hz)**	3112.98	3102.33	180.60	2865.25	3711.3

^1^ VP, voice prosthesis; ^2^ Gy, gray; ^3^ RT, radiotherapy; ^4^ GRBAS scale (grade, roughness, breathiness, asthenia, strain), ^5^ Voice Handicap Index.

**Table 3:** Comparison of the results obtained in the instrumental analysis with the PRAAT and TEVA for the different variables (jitter, shimmer, mean and maximum intensity, fundamental frequency, and harmonic to noise ratio (HNR) obtained with the phonemes [a] and [i] with a microphone and mouth distance between 5 and 10 cm.

	TEVA ^1^			PRAAT			*p*-Value
	Mean ± SD	Median	Range	Mean ± SD	Median	Range	
**[a] Jitter (%)**	2.09 ± 3.34	1.5	0.3–20	2.86 ± 2.61	1.75	0.34–9.19	<0.001
**[a] Shimmer (%)**	25.6 ± 12.8	21.3	8.70–57.3	15 ± 5.86	15.8	0.5–26.3	0.213
**[a] Mean SPL** **(dB)**	64.1 ± 6.22	64	50.6–78.5	63.9 ± 7.08	63.9	51–81	<0.001
**[a] Maximum SPL (dB)**	74.8 ± 10.1	73.1	54.8–91.4	66.9 ± 6.95	66.7	54.9–83.1	<0.001
**[a] Fundamental frequency (F0) (Hz)**	105 ± 41.7	96	49–215	275 ± 83	115	58.4–264	<0.001
**[a] HNR ^2^ (dB)**	3.36 ± 2.12	2.75	0.5–8	3.49 ± 2.36	3.13	0.07–7.99	<0.001
**[i] Jitter (%)**	1.57 ± 1.66	0.85	0.2–6.5	2.8 ± 2.54	1.82	0.29–11.1	<0.001
**[i] Shimmer (%)**	22.4 ± 12.4	17.9	4.1–52.2	14.9 ± 4.27	16	4.83–21.9	<0.001
**[i] Mean intensity** **(dB)**	63.3 ± 5.75	62.5	49.3–76.1	62 ± 6.24	61.7	48.4–74.8	<0.001
**[i] Maximum intensity (dB)**	66.9 ± 6.52	66.8	49.9–80.8	64.8 ± 5.9	64.5	51.1–76.4	<0.001
**[i] Fundamental frequency (F0) (Hz)**	109 ± 47.5	93	59–240	142 ± 117	90.3	54.8–635	<0.001
**[i] HNR (dB)**	5.46 ± 2.67	5.05	0.9–11.1	4.56 ± 2.47	4.2	1.39–10.9	<0.001

^1^ TEVA, Tracheoesophageal Voice Analysis; ^2^ HNR, harmonic to noise ratio.

**Table 4:** Spectrogram analysis with TEVA and PRAAT (Yanagihara classification).

		Grade I	Grade II	Grade III	Grade IV	*p*-Value
		N (%)	N (%)	N (%)	N (%)	
**[a]**	**TEVA**	14 (41.2)	10 (29.4)	4 (11.8)	6 (17.6)	0.201
	**PRAAT**	15 (44.1)	15 (44.1)	3 (8.8)	1 (2.9)	
**[i]**	**TEVA**	8 (23.5)	15 (44.1)	8 (23.5)	3 (8.8)	0.414
	**PRAAT**	13 (38.2)	15 (44.1)	5 (14.7)	1 (2.9)	

## Data Availability

The data presented in this study are available on request from the corresponding author due to privacy.
